# Crystal structure of seleno-l-cystine di­hydro­chloride

**DOI:** 10.1107/S205698901501021X

**Published:** 2015-05-30

**Authors:** Carl Henrik Görbitz, Vladimir Levchenko, Jevgenijs Semjonovs, Mohamed Yusuf Sharif

**Affiliations:** a Department of Chemistry, University of Oslo, PO Box 1033 Blindern, N-0315 Oslo, Norway

**Keywords:** crystal structure, l-cystine analogue, Se—Se bridge, cancer therapy, isotypism

## Abstract

Numerous crystal structures are available for the dimeric amino acid cystine. In proteins it is formed by oxidation of the –SH thiol groups of two closely spaced cysteine residues, resulting in the formation of a familiar di­sulfide bridge. The title compound [systematic name: (*R*,*R*)-1,1′-dicarb­oxy-2,2′-(diselanedi­yl)diethanaminium dichloride], C_6_H_14_N_2_O_4_Se_2_^2+^·2Cl^−^, is the first example of a small mol­ecule structure of the biologically important analogue with a —CH_2_—Se—Se—CH_2_— bridging unit. Bond lengths and angles of seleno-l-cystine di­hydro­chloride and its isotypic sulfur analogue l-cystine di­hydro­chloride are compared.

## Abstract 2

The crystal structure of seleno-l-cystine, in its hydro­chloric acid salt, is isotypic with the common analogue with Se atoms replaced by sulfur, *i.e.* L-cystine hydro­chloride.

## Chemical context   

In addition to the 20 amino acids directly encoded by the genetic code, three more are incorporated into proteins during translation. These three, seleno­cystine, pyrrolysine and *N*-formyl­methio­nine, are considered to belong to a group of 23 proteinogenic amino acids. The UGA codon, normally a stop codon, is made to encode seleno­cysteine by the presence of a seleno­cysteine insertion sequence (SECIS) in the mRNA (Kryukov *et al.*, 2003 ▸).

Analogous to the common sulfur analogue cysteine, seleno­cysteine dimerizes through the formation of an Se—Se bridge to seleno­cystin, a substance that has received considerable attention recently for its anti­cancer efficacy (Yu *et al.*, 2015 ▸) as well as its potential in the prevention of cardiovascular and neurodegenerative diseases (Weekley & Harris, 2013 ▸).
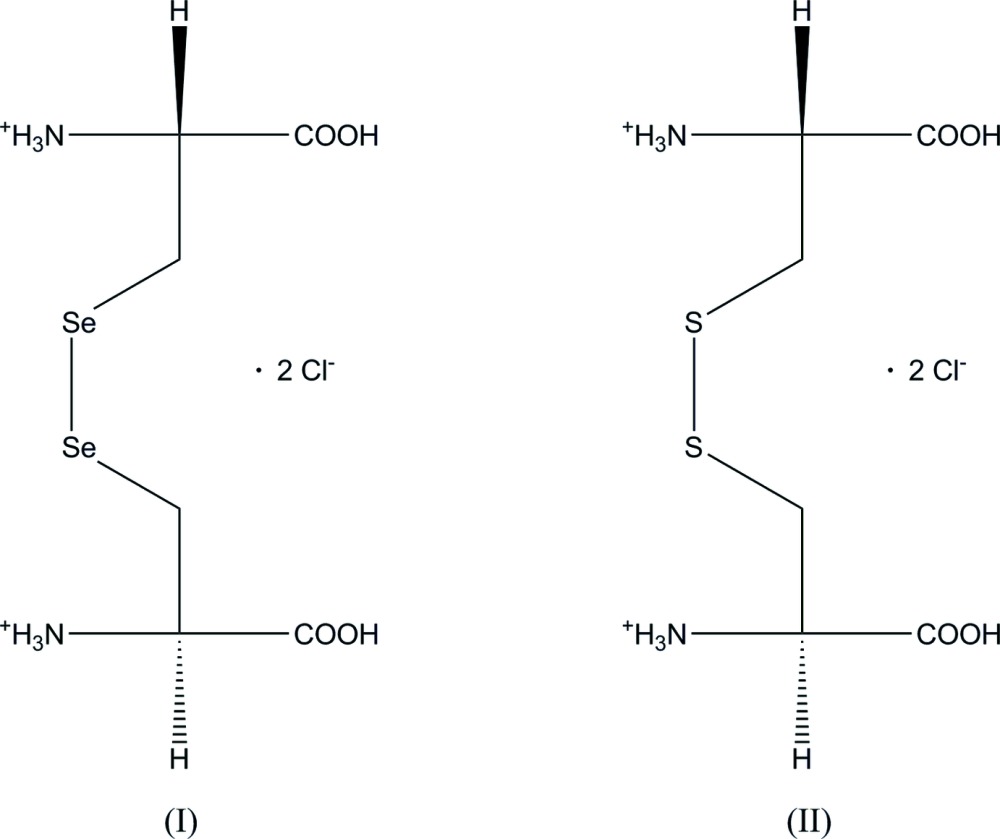


In the Cambridge Structural Database (CSD, version 5.36; Groom & Allen, 2014 ▸) there are about 80 distinct structures of cystine deposited, either as an amino acid, a modified amino acid or as an integrate part of a peptide or another large organic mol­ecule. In contrast, there are no entries for seleno­cystine (and also none for sulfur–selenium hybrids with a —CH_2_—S—Se—CH_2_— bridge). To provide detailed structural information for this biologically important link, an investigation of its structure, in the di­hydro­chloride salt C_6_H_14_N_2_O_4_Se_2_^2+^·2Cl^−^, (I), has been undertaken.

## Structural commentary   

**Figure 1:**
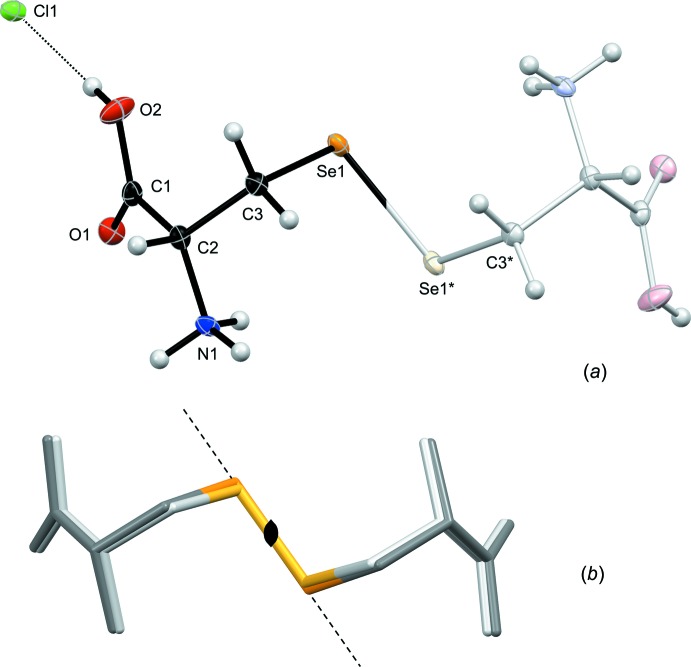
(*a*) The mol­ecular structure of seleno-l-cystine di­hydro­chloride. The right-hand part, coloured in a light tone, is generated by application of twofold rotation symmetry in space group *C*2; Se1*, C3* *etc* are generated by the symmetry code −*x* + 1, *y*, −*z*. Displacement ellipsoids are shown at the 50% probability level. (*b*) Best overlap between the structures of (I) (dark grey O, N and C atoms) and (II) (light grey; Leela & Ramamurthi, 2007 ▸) with a root-mean-square deviation of 0.133 Å. The view is along the twofold rotation axis (lens-shaped symbol), the dashed line gives the direction of the *z* axis.

**Figure 2:**
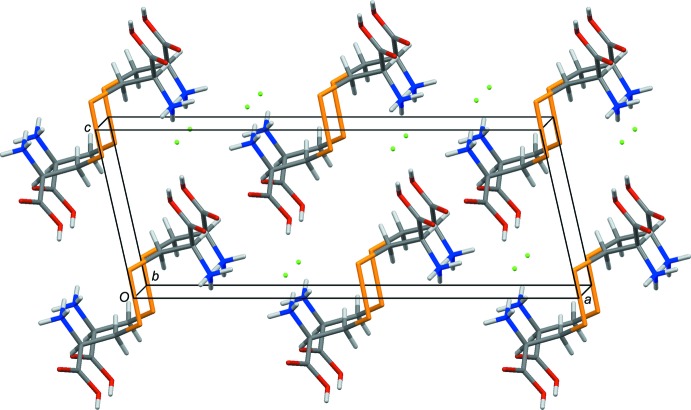
The crystal packing of seleno-l-cystine di­hydro­chloride viewed approximately along the *b* axis.

The mol­ecular structure of (I) is shown in Fig. 1 ▸ *a*. A twofold rotation axis relates the two parts of the mol­ecule. The crystal packing is depicted in Fig. 2 ▸, with mol­ecules stacked on top of each other along the 5.2529 (4) Å monoclinic axis. Compound (I) is isotypic with the structure of l-cystine di­hydro­chloride, (II) (Gupta *et al.*, 1974 ▸; Jones *et al.*, 1974 ▸; Leela & Ramamurthi, 2007 ▸), but not with the structure of l-cystine di­hydro­bromide (Anbuchezhiyan *et al.*, 2010 ▸), which forms a related packing arrangement but crystallizes in the ortho­rhom­bic space group *P*2_1_2_1_2. The di­sulfide/diselenide bridges adopt helical conformations in all three structures, characterized by having *gauche* —C—C—*X*—*X*—, —C—*X*—*X*—C— and —*X*—*X*—C—C— torsion angles (*X* = S or Se) of the same sign, in this case between −81 and −89° [Table 1 ▸; —C—C—*X*—*X*— = —*X*—*X*—C—C— by symmetry]. Geometric parameters for (I) and (II) are furthermore compared in Table 1 ▸ with average values from 16 acyclic —CH_2_—Se—Se—CH_2_— links in non-amino acid structures retrieved from the CSD (Groom & Allen, 2014 ▸). The bond lengths and bond angles of (I) are similar to those in the previous seleno structures. The most important differences with respect to (II) [X-ray data at 173 K: *a* = 18.4405 (15), *b* = 5.2116 (6), *c* = 7.2191 (6) Å, β = 103.856 (6)°; Leela & Ramamurthi, 2007 ▸] are (obviously) the two Se—Se and S—S bond lengths, with modest changes for bond angles and torsion angles. Concerning the dimensions of the unit cell, there is above all an increase in the length of the cell edge *a* (+ 0.364 Å, 2%) due to longer C—Se than C—S bonds. An equivalent, anti­cipated effect on *c* as a result of the increased length of the Se—Se bond, which runs parallel to the *z* axis, is effectively counteracted by a 2.52° decrease for the two C—Se—Se angles along the bridge compared to the C—S—S angles, see: Fig. 1 ▸ *b* and Table 1 ▸. The length of the short monoclinic axis *b* is determined by direct stacking of amino acid mol­ecules, for which the S-to-Se substitution has less impact since neither is involved in any close inter­molecular contacts.

## Supra­molecular features   

**Figure 3:**
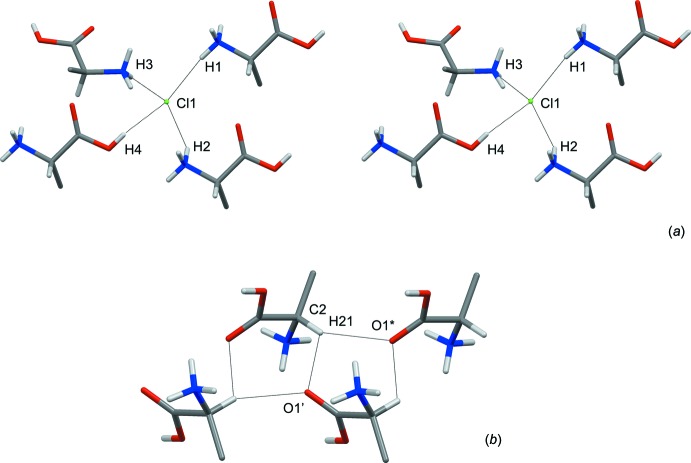
(*a*) Stereodrawing showing the coordination of hydrogen-bond donors around a Cl^−^ anion (see Table 2 ▸ for symmetry operators). (*b*) Tape motif along the *b* axis generated from C^α^—H⋯O hydrogen bonds. O1* is at (*x*, *y* + 1, *z*), O1′ at (−*x* + 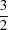, *y* + 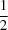, −*z* + 1). Side chains have been truncated beyond C^β^.

The four strong hydrogen bonds with N—H and O—H donors all have Cl^−^ as the acceptor atom (Fig. 3 ▸ *a*). The geometric parameters of the hydrogen bonds listed in Table 2 ▸ are almost identical to those of (II). There is also a three-centre inter­action with a C^α^—H donor and two carbonyl oxygen atoms as acceptors, Fig. 3 ▸ *b*.

## Synthesis and crystallization   

Seleno­cystine has very low solubility in water as well as in organic solvents, including tri­fluoro­ethanol and 1,1,1,3,3,3-hexa­fluoro­propan-2-ol, so a saturated solution was prepared in 0.1 *M* NaOH solution. 100 µl of this solution was pipetted into a small test tube (5 × 50 mm) to which a small amount of BTB pH indicator was added. The tube was sealed with parafilm punctured with a needle (one small hole) and placed inside a larger tube with concentrated hydro­chloric acid. After 15 h the colour had shifted from blue to green, and small crystals of the hydro­chloride could be harvested.

## Refinement   

Crystal data, data collection and structure refinement details are summarized in Table 3 ▸. The position of the carboxyl H atom was restrained to the plane defined by O1, O2, C1 and C2; other H atoms were positioned with idealized geometry with fixed C/N—H distances for NH_3_, CH_2_ (methyl­ene) and CH (methine) groups of 0.91, 0.99 and 1.00 Å, respectively. Free rotation was permitted for the ammonium group. *U*_iso_(H) values were set to 1.2*U*_eq_ of the carrier atom, or 1.5*U*_eq_ for the ammonium group.

A rather large residual peak in the electron density map, with Δρ_max_ = 4.55 e Å^−3^, remained after completion of the refinement. This peak is located on the twofold rotation axis at the center of the Se—Se bond, and evidently reflects bonding electrons. As a test, an extra isotropic C atom was introduced close to the axis. Its occupancy was subsequently refined to 0.17 (equivalent to one electron), and the *R*-factor fell from 0.0233 to 0.0180.

## Supplementary Material

Crystal structure: contains datablock(s) I, global. DOI: 10.1107/S205698901501021X/wm5154sup1.cif

Structure factors: contains datablock(s) I. DOI: 10.1107/S205698901501021X/wm5154Isup2.hkl

Supporting information file. DOI: 10.1107/S205698901501021X/wm5154Isup3.cml

CCDC reference: 1403356

Additional supporting information:  crystallographic information; 3D view; checkCIF report

## Figures and Tables

**Table 1:** Geometric parameters (, ) of diselenide and disulfide bridges

Compound	CSe/CS	SeSe/SS	CCSe/S	CSe/SSe/S	CCSe/SSe/S	CSe/SSe/SC
(I)	1.9671(18)	2.3213(4)	113.96(12)	100.88(5)	88.72(12)	83.05(10)
Average*^*a*^*	1.967	2.310	114.17	101.29		
(II)*^*b*^*	1.817	2.040	114.48	103.40	89.04	81.04

Notes: (*a*) average of 16 CH_2_SeSeCH_2_ bridges in acyclic non-amino acid structures; (*b*) Leela Ramamurthi (2007 ▸).

**Table 2:** Hydrogen-bond geometry (, )

*D*H*A*	*D*H	H*A*	*D* *A*	*D*H*A*
N1H1Cl1^i^	0.91	2.25	3.1425(16)	167
N1H2Cl1^ii^	0.91	2.40	3.2110(18)	149
N1H3Cl1^iii^	0.91	2.32	3.1794(15)	157
O2H4Cl1	0.79(6)	2.22(6)	3.0080(19)	172(4)
C2H21O1^iv^	1.00	2.39	3.292(2)	150
C2H21O1^iii^	1.00	2.55	3.216(2)	124

Symmetry codes: (i) 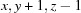; (ii) 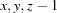; (iii) 
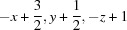
; (iv) 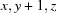.

**Table 3:** Experimental details

Crystal data	
Chemical formula	C_6_H_14_N_2_O_4_Se_2_^2+^2(Cl)
*M*_r_	407.02
Crystal system, space group	Monoclinic, *C*2
Temperature (K)	100
*a*, *b*, *c* ()	18.8045(16), 5.2529(4), 7.2719(6)
()	102.219(1)
*V* (^3^)	702.03(10)
*Z*	2
Radiation type	Mo *K*
(mm^1^)	5.65
Crystal size (mm)	0.85 0.08 0.07

Data collection	
Diffractometer	Bruker D8 Advance single-crystal CCD
Absorption correction	Multi-scan (*SADABS*; Bruker, 2014 ▸)
*T*_min_, *T*_max_	0.514, 1.000
No. of measured, independent and observed [*I* > 2(*I*)] reflections	11089, 4209, 4080
*R*_int_	0.024
(sin /)_max_ (^1^)	0.908

Refinement	
*R*[*F* ^2^ > 2(*F* ^2^)], *wR*(*F* ^2^), *S*	0.023, 0.062, 1.10
No. of reflections	4209
No. of parameters	78
No. of restraints	3
H-atom treatment	H atoms treated by a mixture of independent and constrained refinement
_max_, _min_ (e ^3^)	4.55, 0.87
Absolute structure	Flack *x* determined using 1708 quotients (Parsons *et al.*, 2013 ▸)
Absolute structure parameter	0.044(4)

Computer programs: *APEX2* and *SAINT* (Bruker, 2014 ▸), *SHELXT* (Sheldrick, 2015*a* ▸), *SHELXL2014* (Sheldrick, 2015*b* ▸) and *Mercury* (Macrae *et al.*, 2008 ▸).

## supplementary crystallographic information

### Crystal data

**Table:**

C_6_H_14_N_2_O_4_Se_2_^2+^·2(Cl^−^)	*F*(000) = 396
*M**_r_* = 407.02	*D*_x_ = 1.925 Mg m^−^^3^
Monoclinic, *C*2	Mo *K*α radiation, λ = 0.71073 Å
*a* = 18.8045 (16) Å	Cell parameters from 9938 reflections
*b* = 5.2529 (4) Å	θ = 2.2–40.2°
*c* = 7.2719 (6) Å	µ = 5.65 mm^−^^1^
β = 102.219 (1)°	*T* = 100 K
*V* = 702.03 (10) Å^3^	Needle, colourless
*Z* = 2	0.85 × 0.08 × 0.07 mm

### Data collection

**Table:**

Bruker D8 Advance single-crystal CCD diffractometer	4209 independent reflections
Radiation source: fine-focus sealed tube	4080 reflections with *I* > 2σ(*I*)
Graphite monochromator	*R*_int_ = 0.024
Detector resolution: 8.3 pixels mm^-1^	θ_max_ = 40.2°, θ_min_ = 2.2°
Sets of exposures each taken over 0.5° ω rotation scans	*h* = −34→34
Absorption correction: multi-scan (*SADABS*; Bruker, 2014)	*k* = −9→9
*T*_min_ = 0.514, *T*_max_ = 1.000	*l* = −13→13
11089 measured reflections	

### Refinement

**Table:**

Refinement on *F*^2^	Hydrogen site location: inferred from neighbouring sites
Least-squares matrix: full	H atoms treated by a mixture of independent and constrained refinement
*R*[*F*^2^ > 2σ(*F*^2^)] = 0.023	*w* = 1/[σ^2^(*F*_o_^2^) + (0.0169*P*)^2^ + 0.004*P*] where *P* = (*F*_o_^2^ + 2*F*_c_^2^)/3
*wR*(*F*^2^) = 0.062	(Δ/σ)_max_ < 0.001
*S* = 1.10	Δρ_max_ = 4.55 e Å^−^^3^
4209 reflections	Δρ_min_ = −0.87 e Å^−^^3^
78 parameters	Absolute structure: Flack *x* determined using 1708 quotients (Parsons *et al.*, 2013)
3 restraints	Absolute structure parameter: 0.044 (4)

### Special details

**Table:**

Geometry. All e.s.d.'s (except the e.s.d. in the dihedral angle between two l.s. planes) are estimated using the full covariance matrix. The cell e.s.d.'s are taken into account individually in the estimation of e.s.d.'s in distances, angles and torsion angles; correlations between e.s.d.'s in cell parameters are only used when they are defined by crystal symmetry. An approximate (isotropic) treatment of cell e.s.d.'s is used for estimating e.s.d.'s involving l.s. planes.

### Fractional atomic coordinates and isotropic or equivalent isotropic displacement parameters (Å^2^)

**Table:**

	*x*	*y*	*z*	*U*_iso_*/*U*_eq_	
Se1	0.50147 (2)	0.56610 (5)	0.16037 (2)	0.01306 (4)	
Cl1	0.65288 (2)	0.16979 (8)	0.87546 (6)	0.01345 (7)	
O1	0.69775 (8)	0.3451 (3)	0.4104 (2)	0.0158 (2)	
O2	0.62968 (9)	0.5697 (5)	0.57259 (19)	0.0225 (3)	
H4	0.6356 (18)	0.454 (12)	0.644 (6)	0.058 (15)*	
N1	0.67823 (8)	0.6777 (3)	0.1266 (2)	0.0125 (2)	
H1	0.6681	0.8028	0.0384	0.019*	
H2	0.6563	0.5305	0.0787	0.019*	
H3	0.7272	0.6536	0.1591	0.019*	
C1	0.66257 (8)	0.5306 (3)	0.4320 (2)	0.0125 (3)	
C2	0.65058 (9)	0.7531 (3)	0.2961 (2)	0.0114 (2)	
H21	0.6808	0.8988	0.3574	0.014*	
C3	0.57160 (9)	0.8414 (3)	0.2457 (3)	0.0123 (2)	
H31	0.5667	0.9713	0.1451	0.015*	
H32	0.5592	0.9238	0.3573	0.015*	

### Atomic displacement parameters (Å^2^)

**Table:**

	*U*^11^	*U*^22^	*U*^33^	*U*^12^	*U*^13^	*U*^23^
Se1	0.00843 (6)	0.01357 (7)	0.01686 (7)	−0.00112 (6)	0.00199 (4)	0.00422 (7)
Cl1	0.01334 (15)	0.01271 (15)	0.01538 (15)	0.00216 (12)	0.00550 (12)	0.00565 (12)
O1	0.0175 (6)	0.0106 (5)	0.0193 (6)	0.0028 (4)	0.0037 (5)	0.0040 (4)
O2	0.0349 (7)	0.0201 (6)	0.0146 (5)	0.0104 (8)	0.0100 (5)	0.0067 (7)
N1	0.0104 (5)	0.0118 (5)	0.0159 (5)	0.0005 (4)	0.0045 (4)	0.0045 (5)
C1	0.0125 (5)	0.0113 (7)	0.0126 (6)	0.0005 (4)	0.0000 (4)	0.0034 (4)
C2	0.0114 (6)	0.0083 (5)	0.0138 (6)	0.0003 (5)	0.0011 (5)	0.0021 (5)
C3	0.0125 (6)	0.0096 (5)	0.0147 (6)	0.0022 (5)	0.0029 (5)	0.0019 (5)

### Geometric parameters (Å, º)

**Table:**

Se1—C3	1.9671 (18)	N1—H2	0.9100
Se1—Se1^i^	2.3213 (4)	N1—H3	0.9100
O1—C1	1.206 (2)	C1—C2	1.516 (2)
O2—C1	1.319 (2)	C2—C3	1.525 (2)
O2—H4	0.79 (6)	C2—H21	1.0000
N1—C2	1.490 (2)	C3—H31	0.9900
N1—H1	0.9100	C3—H32	0.9900
			
C3—Se1—Se1^i^	100.88 (5)	N1—C2—C3	112.00 (14)
C1—O2—H4	112 (4)	C1—C2—C3	113.22 (14)
C2—N1—H1	109.5	N1—C2—H21	107.9
C2—N1—H2	109.5	C1—C2—H21	107.9
H1—N1—H2	109.5	C3—C2—H21	107.9
C2—N1—H3	109.5	C2—C3—Se1	113.96 (12)
H1—N1—H3	109.5	C2—C3—H31	108.8
H2—N1—H3	109.5	Se1—C3—H31	108.8
O1—C1—O2	125.92 (18)	C2—C3—H32	108.8
O1—C1—C2	123.24 (17)	Se1—C3—H32	108.8
O2—C1—C2	110.84 (16)	H31—C3—H32	107.7
N1—C2—C1	107.72 (14)		
			
O1—C1—C2—N1	9.3 (2)	N1—C2—C3—Se1	70.66 (16)
O2—C1—C2—N1	−170.64 (15)	C1—C2—C3—Se1	−51.39 (17)
O1—C1—C2—C3	133.72 (17)	C2—C3—Se1—Se1^i^	−88.72 (12)
O2—C1—C2—C3	−46.24 (19)	C3—Se1—Se1^i^—C3^i^	−83.05 (10)

Symmetry code: (i) −*x*+1, *y*, −*z*.

### Hydrogen-bond geometry (Å, º)

**Table:**

*D*—H···*A*	*D*—H	H···*A*	*D*···*A*	*D*—H···*A*
N1—H1···Cl1^ii^	0.91	2.25	3.1425 (16)	167
N1—H2···Cl1^iii^	0.91	2.40	3.2110 (18)	149
N1—H3···Cl1^iv^	0.91	2.32	3.1794 (15)	157
O2—H4···Cl1	0.79 (6)	2.22 (6)	3.0080 (19)	172 (4)
C2—H21···O1^v^	1.00	2.39	3.292 (2)	150
C2—H21···O1^iv^	1.00	2.55	3.216 (2)	124

Symmetry codes: (ii) *x*, *y*+1, *z*−1; (iii) *x*, *y*, *z*−1; (iv) −*x*+3/2, *y*+1/2, −*z*+1; (v) *x*, *y*+1, *z*.
